# Successful treatment with steroid pulse therapy for a COVID-19 case with progressive respiratory failure during treatment for pleural metastasis of breast cancer

**DOI:** 10.1186/s40792-022-01451-4

**Published:** 2022-05-16

**Authors:** Hikari Jimbo, Yoshiya Horimoto, Makoto Hiki, Yoko Tabe, Junichiro Watanabe, Mitsue Saito, Toshio Naito

**Affiliations:** 1 Department of Breast Oncology, Juntendo University Faculty of Medicine, 2-1-1 Hongo, Bunkyo-ku, Tokyo, 113-0033 Japan; 2 Department of Emergency and Disaster Medicine, Juntendo University Faculty of Medicine, 2-1-1 Hongo, Bunkyo-ku, Tokyo, 113-0033 Japan; 3 Department of Cardiovascular Biology and Medicine, Juntendo University Faculty of Medicine, 2-1-1 Hongo, Bunkyo-ku, Tokyo, 113-0033 Japan; 4 Department of Clinical Laboratory Medicine, Juntendo University Faculty of Medicine, 2-1-1 Hongo, Bunkyo-ku, Tokyo, 113-0033 Japan; 5 Department of General Medicine, Juntendo University Faculty of Medicine, 2-1-1 Hongo, Bunkyo-ku, Tokyo, 113-0033 Japan; 6 Department of Research Support Utilizing Bioresource Bank, Juntendo University Graduate School of Medicine, 2-1-1 Hongo, Bunkyo-ku, Tokyo, 113-0033 Japan

**Keywords:** COVID-19, SARS-CoV-2, Steroid pulse therapy, Recurrent breast cancer, Carcinomatous pleurisy

## Abstract

**Background:**

Severe acute respiratory syndrome coronavirus 2 (SARS-CoV-2) infected patients are at high risk for developing severe conditions if other comorbidities are present, such as advanced cancer. Although the regulation of immune response is thought to play an important role in the treatment of coronavirus disease 2019 (COVID-19), physicians often have difficulties in selecting the most appropriate treatment. Furthermore, the impact that interrupting breast cancer treatment due to a COVID-19 infection has on patient outcomes is still unknown. Herein we report a case of advanced breast cancer in a patient whose COVID-19 acute respiratory failure was successfully treated with minimal interruption to their anticancer therapy for recurrent breast cancer.

**Case presentation:**

A 48-year-old woman developed carcinomatous pleurisy after curative surgery for breast cancer. One month after the initiation of targeted therapy with palbociclib and fulvestrant, the pleural effusion decreased, but soon after she developed a COVID-19 infection. Dexamethasone (8 mg/day) was administered due to a prolonged fever, but her respiratory symptoms got worse and pneumonia appeared on a computed tomography (CT) scan 7 days after hospitalization. Thus, steroid pulse therapy (methylprednisolone 1000 mg/day) was administered for 3 days. Her respiratory condition rapidly improved. Two weeks after hospital discharge, complete regression of pneumonia was confirmed on CT scan, and her targeted therapy was resumed at the same dose and strength. More than 6 months later, her metastatic disease remains stable while on the same treatment. Retrospective analysis of the patient's neutralizing antibodies found the neutralizing activity was low in the early stages of infection, but became high after recovery. This suggests the patient acquired an immunity to SARS-CoV-2 through the infection, despite having a mild myelosuppression due to treatment for recurrent breast cancer.

**Conclusions:**

Steroid pulse therapy is available worldwide, and may have an important role in cancer patients who develop severe pneumonia from SARS-CoV-2, by enabling them to avoid any long-term disruption to anticancer therapy. Moreover, it might also be useful when antiviral therapies lose their efficacy due to mutations of the virus, such as the Omicron variant. A critical element in cases such as this one is that treatment decisions are made by a team of specialists, including pulmonologists.

## Background

Severe acute respiratory syndrome coronavirus 2 (SARS-CoV-2) infected patients are at high risk for developing severe conditions, sometimes fatal, with some comorbidities. The Centers for Disease Control and Prevention has categorized cancer as an underlying disease that may place a SARS-CoV-2 patient at risk for developing a more severe condition (https://www.cdc.gov/coronavirus/2019-ncov/need-extra-precautions/index.html). Recent research revealed that patients with cancer who develop coronavirus disease 2019 (COVID-19) remain susceptible to severe outcomes, even after full vaccination [1]. While the control of exaggerated immune response, such as cytokine storm, is thought to be crucial in the treatment of severe COVID-19 [2], the available drugs and current treatment strategies vary across institutions and countries. Thus, in daily clinical situations, it can be difficult for physicians to select the most appropriate treatment.

With the pandemic, many countries have proposed revised triage guidelines in routine breast cancer treatment [3], but the impact that interrupting breast cancer treatment due to a COVID-19 infection has on patient outcomes is still unclear. Herein we report a case of advanced breast cancer in a patient whose COVID-19 acute respiratory failure was successfully treated with minimal interruption to their anticancer therapy.

## Case presentation

**Fig. 1:**
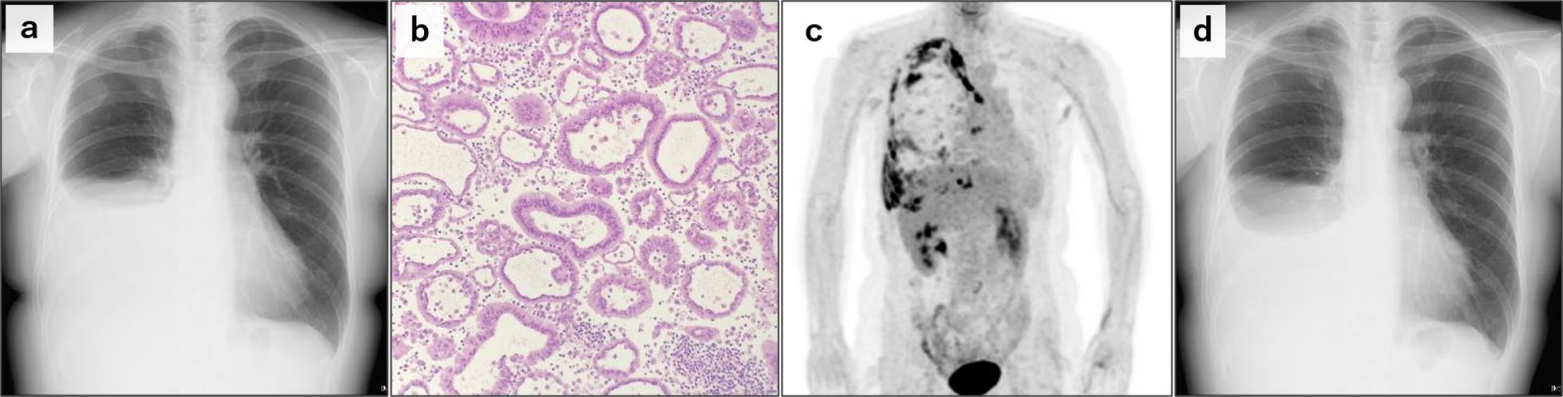
Cytology and imaging of carcinomatous pleurisy. **a** Chest X-ray at the time of recurrence diagnosis. **b** Tubular-forming metastatic breast cancer cells in pleural effusion. **c** Positron emission tomography scan at the time of recurrence diagnosis. **d** Chest X-ray 1 month after the initiation of targeted therapy

A 48-year-old woman with a history of mastectomies for asynchronous bilateral, luminal-type breast cancer had been receiving adjuvant endocrine therapy. Two and a half years after the latest surgery, she felt dyspnea. Chest X-ray revealed a right pleural effusion (Fig. 1a) and, based on cytology, she was diagnosed with carcinomatous pleurisy (Fig. 1b). Image findings from positron emission tomography (PET) at the time of recurrence diagnosis are also shown in Fig. 1c. One month after the initiation of targeted therapy with palbociclib and fulvestrant for recurrent breast cancer, the pleural effusion decreased and her respiratory symptoms improved (Fig. 1d).

**Fig. 2:**
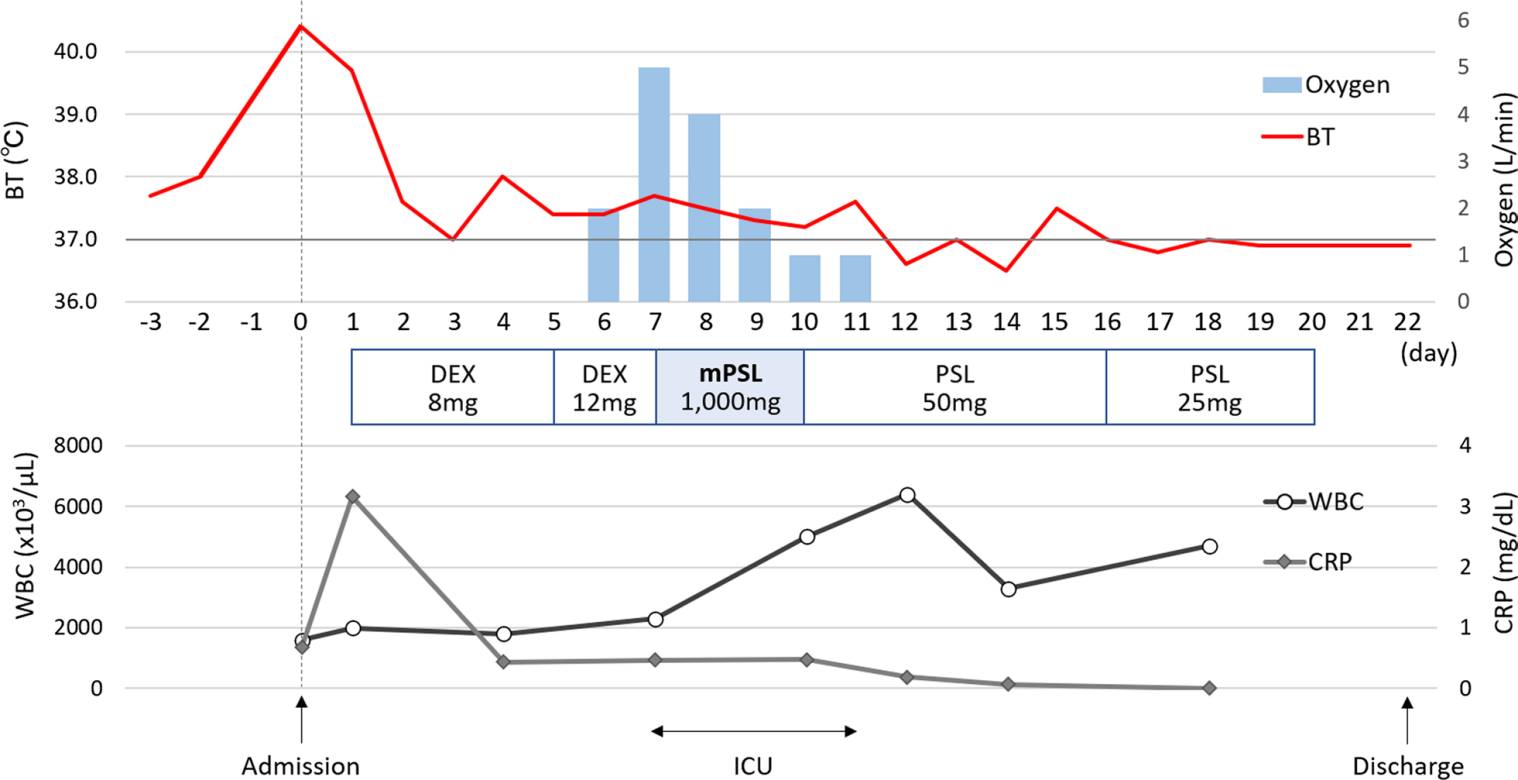
Clinical course of the patient. BT: body temperature; WBC: white blood cell; CRP: C-reactive protein; ICU: intensive care unit; DEX: dexamethasone; mPSL: methylprednisolone

On day 14 of cycle 2 of palbociclib plus fulvestrant therapy, a family member living with her was diagnosed with COVID-19. The next day, the patient developed fever and cough, and a COVID-19 infection was confirmed by polymerase chain reaction (PCR) test 2 days later. At that time in Japan, vaccination against COVID-19 had been completed for only medical professionals and some elderly people. At the time of COVID-19 diagnosis, the patient did not develop overt pneumonia and oxygen supply was not required, with peripheral oxygen saturation (SpO_2_) of 96%. Laboratory testing revealed a slight elevation of C-reactive protein (CRP) at 0.68 mg/dl. Mild neutropenia of 1.15 × 10^9^/l and lymphopenia of 0.42 × 10^9^/l associated with palbociclib therapy was observed, and drugs for recurrent disease were discontinued at the time of hospitalization. The clinical course of the patient after hospital admission is shown in Fig. 2.

**Fig. 3:**
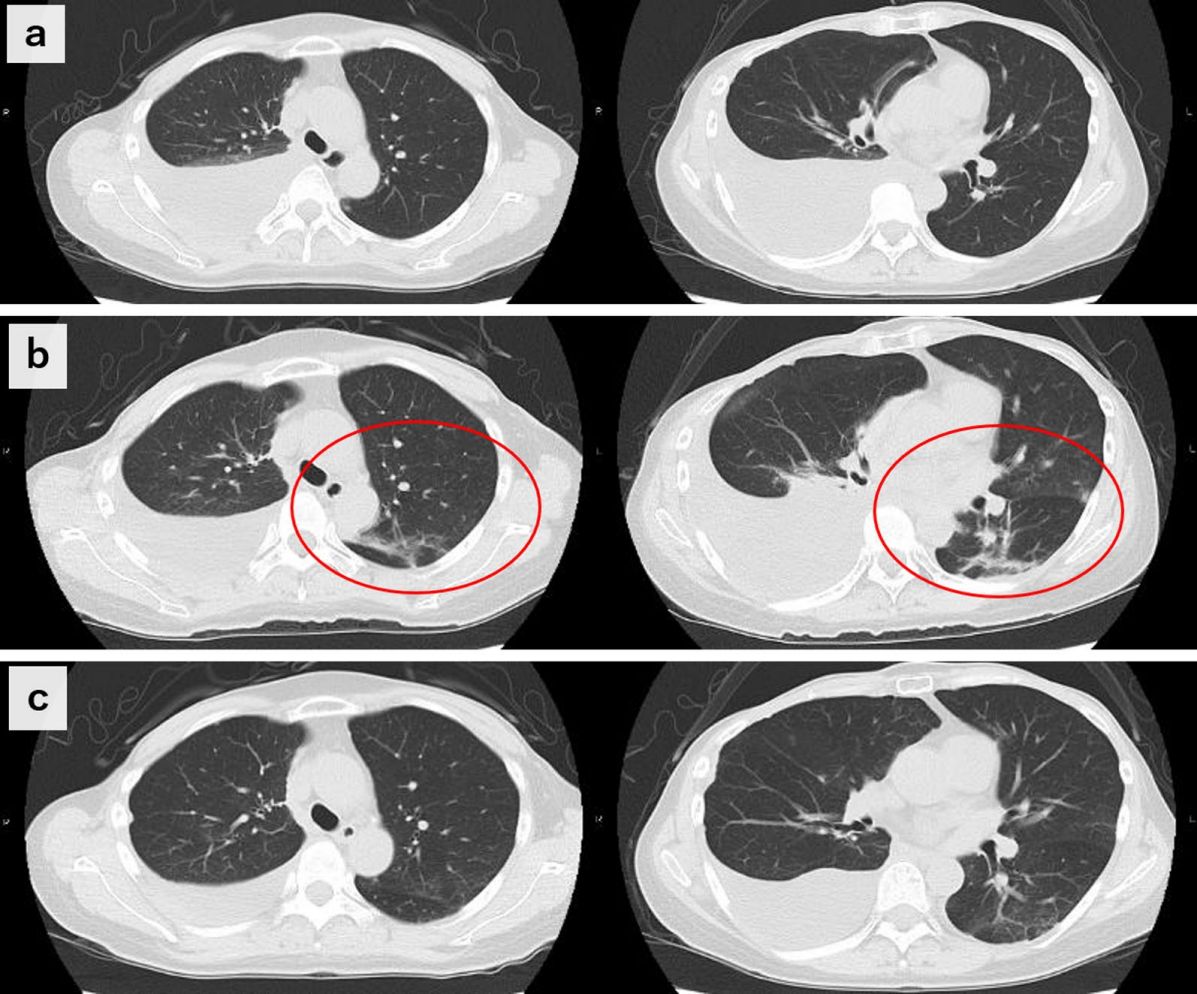
CT scan findings over the clinical course. **a** Day of hospitalization; **b** 7 days after hospitalization; **c** 2 weeks after hospital discharge. Red circles indicate pneumonia

During hospitalization, the patient was treated by a team of internal medicine specialists, including pulmonologists. At admission, the case presented with fever that prolonged until the day after admission. As such, dexamethasone at 8 mg/day was administered, but the patient’s condition was not improved. On the seventh day after hospitalization, her SpO_2_ decreased to 91%, and pneumonia appeared on a computed tomography (CT) scan (Fig. 3a and b). Thus, she was moved to the intensive care unit (ICU) and administered 3 days of steroid pulse therapy with methylprednisolone (mPSL) at 1000 mg/day. Her respiratory condition then improved rapidly, and she was discharged 22 days after admission without relapse, even after tapering off steroids. Two weeks after discharge, a follow-up CT scan showed complete regression of the pneumonia (Fig. 3c), with further decrease in the pleural effusion. At this point, targeted therapy was resumed at the same dose and strength, and the patient has maintained stable disease for more than 6 months.

After obtaining the patient's informed consent, we retrospectively measured the patient's paired neutralizing antibody titers against SARS-CoV-2 (Table 1). The analytical and clinical performance of the assay have been evaluated and are described elsewhere [4]. The c-pass, which measures neutralizing activity and is a direct indicator of the presence of neutralizing antibodies, was low in the early stages of infection, but became high after recovery, and both N- and S-antibodies also became positive. The patient had a mild myelosuppression due to treatment for recurrent breast cancer, but seemed to acquire immunity to SARS-CoV-2 through the infection.

**Table 1:** Changes in neutralizing antibodies against SARS-CoV-2

Date collected	C-pass^*^	IgG (N antibody)^**^	Decision	IgGII (S antibody)^***^	Decision
Day 0	32.8%	0.07 Index	–	2.5 AU/ml	–
Day 1	31.9%	0.11 Index	–	0.8 AU/ml	–
Day 150	96.7%	6.78 Index	+	8072.3 AU/ml	+

^*^ < 30% negative, ≥ 30% positive; ^**^ < 1.0 negative, ≥ 1.0 positive; ^***^ < 50AU/ml negative, ≥ 50AU ml positive

## Discussion

The role of mPSL for COVID-19 patients has not yet been established. As of early 2020, there was a lack of evidence for the efficacy of treatment with corticosteroids [5]. However, the usefulness of mPSL for acute respiratory distress syndrome (ARDS) in terms of improving patient outcomes has been reported [6]. Moreover, some case reports support the efficacy of mPSL pulse therapy in patients with COVID-19 pneumonia [7, 8]. Our observations indicate mPSL pulse therapy should be considered as a treatment option in patients with advanced solid tumors who are being treated with myelosuppressive drugs and develop symptomatic COVID-19-associated pneumonia, in order to avoid any long-term disruption to their anticancer therapy.

Novel antiviral therapies for SARS-CoV-2 infection, such as casirivimab/imdevimab cocktail or remdesivir, have rapidly been introduced, mainly in developed countries. However, their availability is limited due to the evidence level and cost. mPSL pulse therapy on the other hand, is relatively cheap and available worldwide.

There is also the possibility that the usefulness of mPSL pulse therapy is even greater when current antiviral therapies lose their efficacy due to mutations in the virus. For instance, in response to the recent spread of the Omicron variant, the National Institutes of Health stated that “when the Omicron variant represents the majority (e.g., > 80%) of infections in a region, it is expected that bamlanivimab plus etesevimab and casirivimab plus imdevimab will not be active for treatment or post-exposure prophylaxis (PEP) of COVID-19.” (https://www.covid19treatmentguidelines.nih.gov/therapies/statement-on-anti-sars-cov-2-mabs-and-rdv-and-omicron/).

## Conclusions

We were able to save a patient with respiratory failure due to COVID-19 with an underlying cancerous pleural effusion, enabling her to successfully resume treatment for recurrent breast cancer. Treatment by a team of internal medicine specialists, including respiratory physicians, was a critical element to the success of this case, and while this is just a single case, we hope that it will be a useful example for other medical professionals in daily practice.

## Data Availability

Not applicable.

## Abbreviations

ARDS: Acute respiratory distress syndrome
CT: Computed tomography
CRP: C-reactive protein
ICU: Intensive care unit
PET: Positron emission tomography
WBC: White blood cell
